# Histidine in Health and Disease: Metabolism, Physiological Importance, and Use as a Supplement

**DOI:** 10.3390/nu12030848

**Published:** 2020-03-22

**Authors:** Milan Holeček

**Affiliations:** Department of Physiology, Faculty of Medicine in Hradec Králové, Charles University, Šimkova 870, 500 38 Hradec Kralove, Czech Republic; holecek@lfhk.cuni.cz

**Keywords:** histidine supplementation, HTK solution, carnosine, beta-alanine, ammonia, glutamine, branched-chain amino acids, Bretschneider’s solution

## Abstract

L-histidine (HIS) is an essential amino acid with unique roles in proton buffering, metal ion chelation, scavenging of reactive oxygen and nitrogen species, erythropoiesis, and the histaminergic system. Several HIS-rich proteins (e.g., haemoproteins, HIS-rich glycoproteins, histatins, HIS-rich calcium-binding protein, and filaggrin), HIS-containing dipeptides (particularly carnosine), and methyl- and sulphur-containing derivatives of HIS (3-methylhistidine, 1-methylhistidine, and ergothioneine) have specific functions. The unique chemical properties and physiological functions are the basis of the theoretical rationale to suggest HIS supplementation in a wide range of conditions. Several decades of experience have confirmed the effectiveness of HIS as a component of solutions used for organ preservation and myocardial protection in cardiac surgery. Further studies are needed to elucidate the effects of HIS supplementation on neurological disorders, atopic dermatitis, metabolic syndrome, diabetes, uraemic anaemia, ulcers, inflammatory bowel diseases, malignancies, and muscle performance during strenuous exercise. Signs of toxicity, mutagenic activity, and allergic reactions or peptic ulcers have not been reported, although HIS is a histamine precursor. Of concern should be findings of hepatic enlargement and increases in ammonia and glutamine and of decrease in branched-chain amino acids (valine, leucine, and isoleucine) in blood plasma indicating that HIS supplementation is inappropriate in patients with liver disease.

## 1. Introduction and Aims

L-Histidine (HIS) is a nutritionally essential amino acid (EAA) with unique biochemical and physiological properties, which have created a good theoretical rationale to suggest the use of HIS as a nutritional supplement in a wide range of conditions. Initially, HIS was shown to treat rheumatoid arthritis and anaemia in patients with chronic renal failure [1,2]. Currently, HIS and/or HIS-containing dipeptides (HIS-CD) are investigated to prevent fatigue during strenuous exercise and for therapy in ageing-related disorders, metabolic syndrome, atopic dermatitis, ulcers, inflammatory bowel diseases, ocular diseases, and neurological disorders [3,4,5,6,7,8,9].

The first aim of the article is to provide an overview of main pathways of HIS metabolism; chemical and biological properties of HIS, such as proton buffering, metal ion chelation, and antioxidant functions; and a role of several proteins and peptides containing large amounts of HIS residues, such as carnosine (CAR), filaggrin, and histatins. With this explanation as a background, the results of studies examining the benefits and therapeutic potential of HIS and HIS-CD will be discussed or reviewed. 

## 2. Histidine, Chemical, and Biological Properties

**Figure 1:**
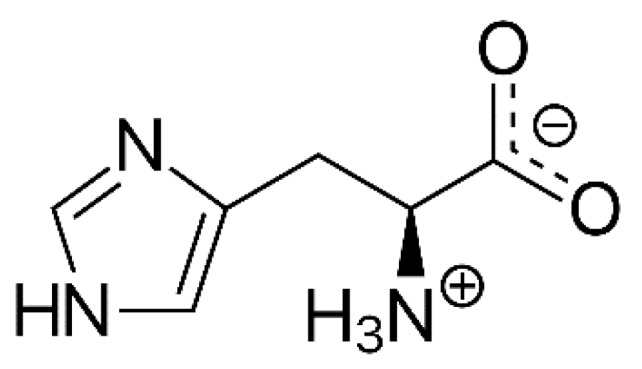
Histidine structure: histidine (HIS) contains an α-amino group, a carboxylic acid group, and an imidazole side chain. Under physiological conditions, the amino group is protonated and the carboxylic group is deprotonated. The imidazole ring is responsible for the proton buffering, metal ion chelating, and antioxidant properties.

The unique chemical properties of HIS, which are mainly attributed to the imidazole ring (Figure 1), include proton buffering, metal ion chelation, and antioxidant activities. These cytoprotective interactions may involve free HIS, HIS-containing peptides, HIS-CD, and HIS residues in proteins.

### 2.1. HIS as a pH Buffer

Of all the amino acid side chains in proteins, only the imidazole ring of HIS is suitable to function as a pH buffer [10], and either of the two nitrogens of the imidazole ring can bind or release a proton to form the acid or the base form. The pKa values of imidazole group of free L-HIS are 6.2 and 6.5 when bound in proteins, 7.0 in CAR, and 7.1 in anserine [11]. Therefore, HIS-CD, such as CAR and anserine, act as powerful buffers and attenuate changes in intracellular pH in muscles during anaerobic exercise [11]. The role of HIS as an efficient H^+^ buffer enables use of HIS as a component of solutions employed for organ preservation before transplantation and myocardial protection in cardiac surgery [12,13,14].

### 2.2. HIS and Metal Ion Chelation

Several studies have reported the ability of HIS and HIS-CD, particularly CAR, and HIS-rich proteins to form complexes with metal ions, such as Fe^2+^, Cu^2+^, Co^2+^, Ni^2+^, Cd^2+^, and Zn^2+^ [15,16]. Specifically, HIS is responsible for binding of iron in haemoglobin and myoglobin molecules and is frequently present in the active sites of metalloenzymes, such as carbonic anhydrase, cytochromes, heme peroxidases, nitric oxide synthase, and catalases, where plays a role in regulating their activity. Histidine-rich glycoprotein present in plasma of vertebrates interacts with many ligands, including zinc, has an important role in immunity [15].

Several metal ions promote the production of free radicals through the Fenton reaction [17] and exert toxic effects on organism, which can be attenuated by HIS or HIS-CD. It has been proven that CAR protects against copper- and zinc-induced neurotoxicity [18].

### 2.3. HIS as an Antioxidant

The antioxidant activity of HIS is mediated by metal ion chelation (see above), by the scavenging of reactive oxygen (ROS) and nitrogen (RNS) species, and by sequestering advanced glycation (AGE; e.g., glyoxal and methylglyoxal) and advanced lipoxidation (ALE; e.g., malondialdehyde and acrolein) end products [19,20,21,22,23,24]. High concentrations of AGE/ALE are recognized as noxious factors related to various complications, notably, microangiopathy and retinopathy of diabetes [23].

HIS-CD, particularly CAR, is more effective ROS/RNS and AGE/ALE scavengers than free HIS [19,20]. The underlying mechanisms of the antioxidant effects of imidazole-containing compounds remain obscure [25].

## 3. HIS Requirements and Sources

### 3.1. Effects of a HIS-Deficient Diet

HIS-deficient diet does not result in the prompt negative protein balance observed with other EAAs. Therefore, HIS was originally classified as a dispensable, nonessential amino acid [26]. Then, the body has been shown to compensate for a HIS deficiency in food for long periods through the enhanced catabolism of haemoglobin and CAR followed by a decrease in the haemoglobin levels in the blood and CAR content in the muscles [27,28,29,30,31]. The evidence of HIS essentiality to maintain positive nitrogen balance was shown in rats by Nasset and Gatewood [32] and in adult humans fed a HIS-deficient diet for at least one moth [27,28,29,30,31]. In addition, atopic dermatitis and decreased HIS levels in plasma and urine are frequently observed is HIS-deficient subjects [27].

### 3.2. Requirements and Sources of Dietary HIS

Estimated average requirement and recommended dietary allowance for HIS are 11 mg/kg/day and 14 mg/kg/day, respectively, for adults of 19 years and older [32]. HIS is obtained from the diet mainly in the form of proteins. Its content in proteins of animal sources, like meat, chicken, and fishes, is 25–30 mg/g, and in plant proteins, like soybean, kidney beans, peas, oat, and wheat, is 20–30 mg/g [33]. Animal sources are better due to higher content of proteins.

High amounts of HIS (109 mg/g) have been detected in dried bonito broth, a food ingredient used commonly in Japanese meals, called dashi. Bonito (skipjack tuna; *Katsuwonus pelamis*) is commonly consumed fish; dried bonito broth is used more frequently than beef or chicken bouillon for soup stock [34].

In addition to proteins, HIS is present in the mammalian musculature as part of HIS-CD, notably CAR and anserine. A rich source of CAR and anserine is a chicken breast extract (CBEX^TM^) used mainly in Japan. CBEX^TM^ is obtained via hot-water extraction of chicken breast, anserine content is ~1.4 g/100 mL, and CAR content is ~0.6 g/100 mL [35]. Both dashi and CBEX^TM^ have been used in several studies examining the effects of HIS and HIS-CD supplementation [9,36,37].

It should be noted that cellular concentrations of HIS and HIS-related compounds in specific organs relate to their functions. For example, high concentrations of CAR and anserine are found in muscles (buffering/antioxidant role) and high concentrations of N-acetyl-L-HIS are found in brain, retina, and lens of poikilothermic vertebrates (osmolyte/antioxidant role) [38]. Concentrations of CAR are higher in fast-twitch (white) muscles when compared with slow-twitch (red) muscles; in the case of HIS concentrations, the opposite is true [39].

## 4. HIS Metabolism

**Figure 2:**
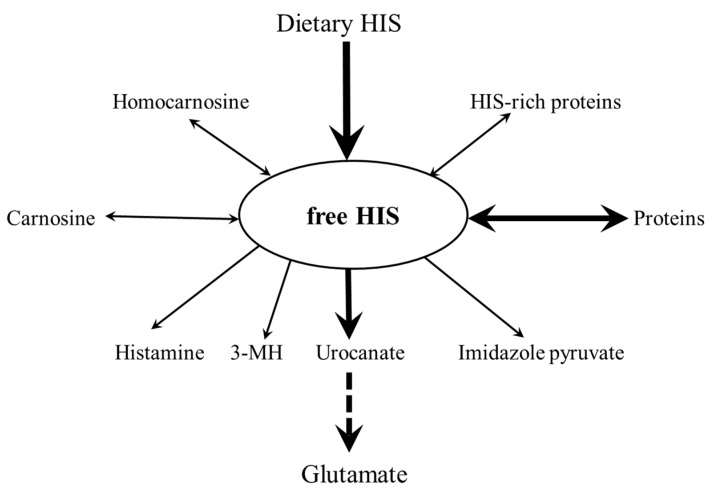
Main pathways of HIS metabolism. Most HIS metabolism is directed to protein turnover and catabolism to glutamate. The minor pathways, such as synthesis of carnosine (CAR), histamine, and HIS-rich proteins, make HIS unique among other amino acids.

There are several pathways of HIS metabolism (Figure 2). Quantitatively most significant are HIS turnover in synthesis and breakdown of proteins and HIS catabolism via urocanate to glutamate. I will overview the pathways of HIS catabolism and importance of HIS as a precursor of histamine, HIS-rich proteins, HIS-containing dipeptides (particularly CAR), and methyl- and sulphur-containing derivatives of HIS.

### 4.1. Catabolism of HIS

**Figure 3:**
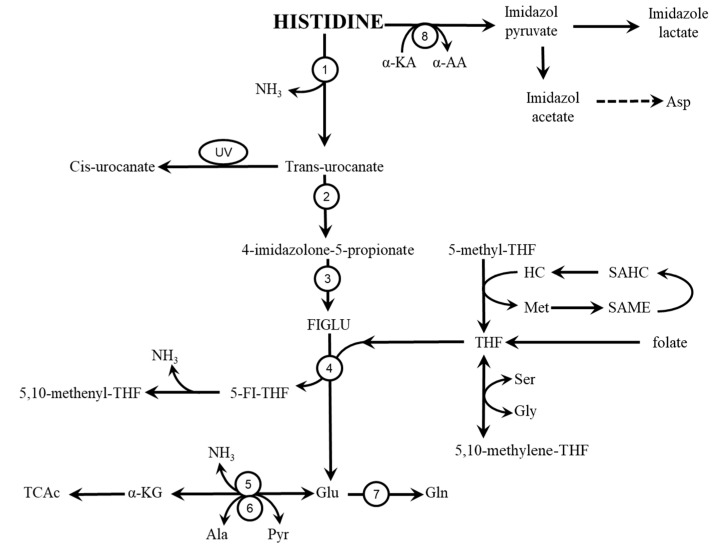
HIS catabolism. 1, histidase; 2, urocanase, 3, imidazolone propionate hydrolase; 4, glutamate formimino transferase; 5, glutamate dehydrogenase; 6, alanine aminotransferase; 7, glutamine synthetase; 8, histidine aminotransferase. Ala, alanine; Asp, aspartic acid; FIGLU, formiminoglutamate; Gln; glutamine; Glu, glutamic acid; Gln, glutamine; Gly, glycine; HC, homocysteine; Met, methionine; Pyr, pyruvate; SAHC, S-adenosylhomocysteine; SAME, S-adenosylmethionine; Ser, serine; THF, tetrahydrofolate; TCAc, tricarboxylic acid cycle; UV, ultraviolet radiation; α-AA, α-amino acid; α-KA, α-keto acid; α-KG, α-ketoglutarate.

Average daily intake of HIS of about 800 mg in adult humans implies that the same amount of HIS should be degraded. The main pathway of HIS catabolism (Figure 3) begins with deamination catalysed by histidase (EC 4.3.1.3), leading to the production of *trans*-urocanate and ammonia. The enzyme is primarily located in the stratum corneum of the skin and the liver.

#### 4.1.1. HIS Catabolism in the Skin

In the skin, filaggrin, a skin barrier protein with high HIS content, is the main HIS source for histidase to generate ammonia and urocanate [40]. Because most of the ammonia produced in the splanchnic region is detoxified to urea in the liver, the skin should be considered a significant source of blood ammonia in the systemic circulation.

Since the skin lacks urocanase (the second enzyme in HIS catabolism), *trans*-urocanate accumulates in the stratum corneum, contributing to the formation of “natural moisturizing factors”, and acts as one of the major ultraviolet (UV)-absorbing compounds [40]. In the presence of UV radiation, *trans*-urocanate is isomerized to *cis*-urocanate, which probably plays a role in the UV radiation-induced suppression of the immune system [41].

#### 4.1.2. HIS Catabolism in the Liver

Histidase expression in the liver is regulated by HIS availability. Histidase activity increases when protein intake is high and decreases when protein intake is low [42,43]. Urocanase (EC 4.2.1.49) converts the urocanate produced in the liver by a histidase reaction to imidazolone propionic acid, which is hydrolysed to formiminoglutamate (FIGLU). FIGLU is converted to glutamic acid by formimino transferase (E.C. 2.1.2.5) in a tetrahydrofolate (THF)-dependent reaction. If folate is deficient, FIGLU accumulates and HIS catabolism is impaired [44]; HIS-loading (FIGLU excretion test) is a diagnostic tool for THF deficiency [45]. Depletion of THF after HIS loading may cause a net reduction in the capacity for glycine synthesis from serine (see Figure 3), as described by Meléndez-Hevia et al. [46] and by Holeček and Vodeničarovová [39].

THF is derived from several sources that might affect the flux of HIS through the HIS degradation pathway (Figure 3). Several articles have demonstrated that methionine, S-adenosylmethionine, homocysteine, and S-adenosylhomocysteine activate HIS catabolism by increasing the availability of THF [47,48].

Glutamate produced by the formimino transferase reaction may be used for synthesis of glutamine, may become deaminated to α-ketoglutarate in a glutamate dehydrogenase reaction, and/or may be released to the blood [49]. Transamination to alanine is unlikely to occur due to excess alanine concentration obtained from extrahepatic tissues.

A high HIS concentration increases HIS flux through the HIS degradation pathway, resulting in increased ammonia production and altered concentrations of several amino acids, particularly increased concentrations of glutamate, alanine, and glutamine and decreased branched-chain amino acids (BCAA) concentrations in the blood plasma [39].

#### 4.1.3. Role of HIS Aminotransferase

The minor pathway of HIS degradation is mediated by HIS aminotransferase, which transforms HIS to imidazole pyruvate, leading to aspartate production (Figure 3). HIS aminotransferase exists in two isoforms. Isoenzyme 1 is expressed only in the liver and is active towards pyruvate and α-ketoglutarate. Isoenzyme 2 is expressed in the liver, kidneys, heart, and skeletal muscle and is active towards pyruvate (resulting in alanine formation) and not active towards α-ketoglutarate [50].

### 4.2. Histamine

**Figure 4:**
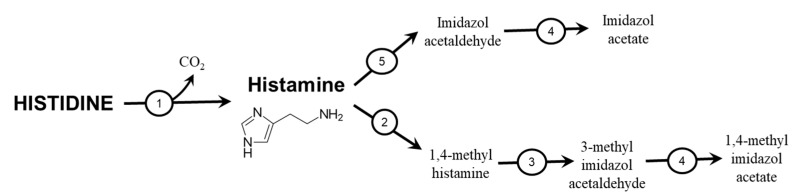
Synthesis and degradation of histamine: Histamine is formed by the decarboxylation of HIS by L-histidine decarboxylase (EC 4.1.1.22) found in many tissues. Released histamine is degraded to 1,4-methyl imidazoleacetic acid; the second pathway of histamine degradation is oxidation to imidazoleacetic acid. The metabolites are released in the urine or processed to other metabolites. 1, histidine decarboxylase; 2, histamine-N-methyltransferase; 3, monoamino oxidase; 4, aldehyde dehydrogenase; 5, diamino oxidase.

Most histamine is synthesized and stored in granules in mast cells and basophils, from which it is released via degranulation induced by immunological stimulation (Figure 4), particularly interactions of allergens with IgE antibodies. Parietal cells in the stomach and histaminergic neurons in the brain are additional important sites of histamine synthesis and storage. Parietal cells produce hydrochloric acid; histaminergic neurons of the posterior hypothalamus modulate a variety of physiological functions, including appetite, wakefulness, emotions, and cognitive functions.

Histamine plays also an important role as a regulator of microcirculation in muscles during exercise and sustained post-exercise vasodilation [51,52]. Histidine decarboxylase expression is induced in mast cells, vascular endothelial cells, and muscle fibers themselves by cytokines (particularly IL-1 and TNF-α), increased temperature, decreased pH, and hypoxia-inducible factor 1 [53,54,55,56].

Histamine exerts its effects through four types of G protein-coupled receptors: H1, H2, H3, and H4 (Table 1) [57]:

#### Effects of Dietary HIS on Histamine Levels

According to several studies, dietary HIS affects histamine concentrations in immune cells, the stomach, and the brain [58,59,60,61]. Altered function of the immune system, allergic reactions, and/or peptic ulcers have not been reported after HIS administration. However, HIS administration has been shown to affect brain function [62,63,64,65,66,67,68].

Increased HIS intake in the form of a dried bonito broth has improved mood state and mental task performance of human subjects [33,34,36]. Several studies have reported an anorectic effect of HIS administration [65,66,67,68] and HIS-enriched diet [62,63,64]. Insufficient HIS intake reduces the brain histamine content and is associated with anxiety-like behaviors in mice [61].

### 4.3. Methyl- and Sulphur-Containing Derivatives of HIS

**Figure 5:**
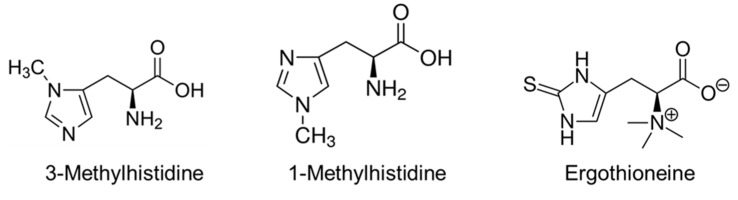
Methyl- and sulphur-containing derivatives of HIS.

The major HIS derivatives present in the human body are 3-methylhistidine, 1-methylhistidine, and ergothioneine (Figure 5).

#### 4.3.1. 3-Methylhistidine (3-MH)

3-MH is formed by the posttranslational methylation of HIS residues of the main myofibrillar proteins actin and myosin. During protein catabolism, 3-MH is released but cannot be reutilized. Therefore, the plasma concentration and urine excretion of 3-MH are sensitive markers of myofibrillar protein degradation [69]. Approximately 75% of 3-MH is estimated to originate from skeletal muscle [69]. In addition to the degradation of muscle proteins, the 3-MH level is affected by the degradation of intestinal proteins and meat intake.

#### 4.3.2. 1-Methylhistidine (1-MH)

*1-MH* is not formed in humans and results from the metabolism of the dipeptide anserine obtained from food. 1-MH represents a potentially useful marker of meat intake. In the absence of meat or fish in the diet, the excretion of 1-MH is minimal and predicts a vegetarian status [70].

#### 4.3.3. Ergothioneine

Ergothioneine (2-mercapto-L-histidine trimethyl-betaine) contains a sulphur atom on the imidazole ring. It is produced from HIS by cyanobacteria, mycobacteria, and fungi. In humans, ergothioneine is acquired from the diet and accumulates in many tissues. The precise physiological role of ergothioneine remains unclear.

It has been hypothesized that ergothioneine administration may prevent tissues against oxidative damage [71] and that decreased blood plasma levels of ergothioneine have been reported in elderly [72] and Parkinson’s disease [73]. Studies in animals and humans have found no toxicity, and ergothioneine has been recently approved as a nutritional supplement [74,75].

### 4.4. HIS-Rich Proteins and Peptides

The main HIS-rich proteins include haemoproteins, HIS-rich glycoprotein, histatins, HIS-rich calcium-binding protein, and filaggrin (Table 2).

### 4.5. HIS-Containing Dipeptides (HIS-CD)

**Figure 6:**
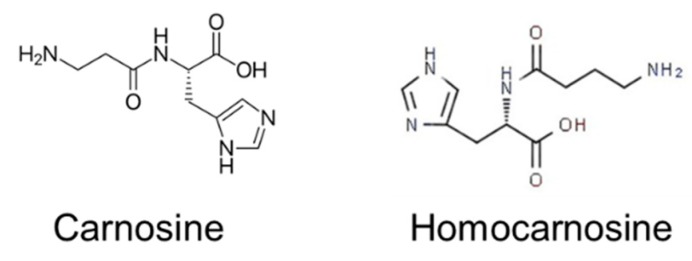
Carnosine and homocarnosine.

The main HIS-CD synthetized in humans are CAR (beta-alanyl-L-histidine) and homocarnosine (gamma-aminobutyryl-L-histidine) (Figure 6).

#### 4.5.1. L-Carnosine (CAR)

CAR is synthesized under hydrolysis of ATP from HIS and beta-alanine, which is obtained through the diet or uracil degradation in the liver [10]. CAR is abundantly present in skeletal muscle and olfactory bulb and in smaller quantities in the cardiac muscle, brain, and other tissues [11,16,79].

CAR is an efficient intracellular pH buffer, heavy metal chelator, anti-glycating agent, and regulator of many receptors [11]. Increased muscle CAR concentrations are presumed to exert ergogenic effects and to decrease fatigue during high-intensity exercise [80]. Fast-twitch muscle fibres have, in accordance with their anaerobic energy delivery and supposed role of CAR as a pH buffer, higher CAR content compared with slow-twitch fibres [39,81]. In experiments with rapidly ageing mice, CAR delayed the ageing of the animals, probably due to the improvement in their antioxidant status [22].

CAR is catabolised by 2 enzymes [82,83]. The enzyme carnosinase (CN1) exhibits narrow specificity and is present in the serum and brain. The enzyme CN2 (also known as a nonspecific dipeptidase) exhibits broad substrate specificity and is ubiquitously expressed. It has been shown that most of the CAR provided by food is rapidly hydrolysed by serum carnosinase to HIS and beta-alanine, which can then be taken up by muscles where CAR is synthesized [84].

#### 4.5.2. Homocarnosine

Homocarnosine has been detected in the brain, but its physiological function has not been completely elucidated. Homocarnosine has been suggested to serve as a source of gamma-aminobutyric acid (GABA), the main inhibitory neurotransmitter in the mammalian brain [85].

#### 4.5.3. Other HIS-CD

Other HIS-CD found in vertebrates and not in invertebrates, plants, and fungi include anserine (beta-alanyl-N-π-methylhistidine), balenine (ophidine, beta-alanyl-N-tau-methylhistidine), acetyl carnosine (N-acetyl-β-alanyl-L-histidine), carcinine (beta-alanylhistamine), and homoanserine (gamma-aminobutyryl-L-1-histidine). A dipeptide, HIS-leucine, forms in the process of converting angiotensin I to angiotensin II, and this dipeptide does not appear to exert any haemodynamic effects in normotensive and hypertensive rats [86].

## 5. HIS and HIS-Containing Substances as Nutritional Supplements

**Figure 7:**
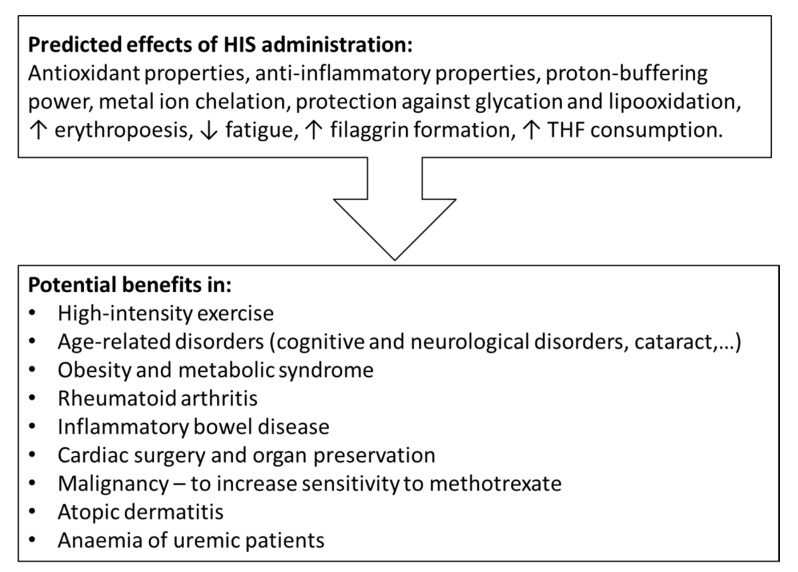
Predicted effects and potential benefits of HIS-containing supplements.

Due to the wide range of potentially beneficial physiological properties, such as antioxidant properties, proton-buffering power, and chelating function, HIS-containing supplements have been investigated in the wide range of conditions (Figure 7). In most studies, daily HIS supplementation doses range from 1 to 4 g, which represents approximately 2–8% of the recommended intake of nitrogen and an increase in daily intake of HIS up to six times [3,34,87].

CAR is predicted to be a more efficient proton-buffering and antioxidant compound than HIS. Hence, several intervention studies have been performed using CAR, which is rapidly inactivated by serum carnosinase in humans [84]. Therefore, short-term studies indicate possibly the combined effects of HIS and beta-alanine rather than CAR. If it concerns chronic supplementation interventions, these studies can indicate effects of muscle CAR loading.

### 5.1. Effects on Muscle Performance and Fatigue

HIS supplementation is predicted to increase the intracellular CAR concentration, which effectively buffers hydrogen ions formed during high-intensity exercise and might ameliorate fatigue due to increased histamine synthesis in the brain [34,36,37]. However, it has been shown that the rate-limiting precursor of CAR synthesis in humans is beta-alanine and that its chronic supplementation is more effective at increasing the CAR content than HIS [81,88,89,90]. Beta-alanine administered in daily doses of 4.8–6.4 g increased human muscle CAR content by 60% in 4 weeks and 80% in 10 weeks [80,91].

Several original and review articles have described the positive effects of long-term beta-alanine supplementation on muscle performance [92,93,94,95,96], and chronic beta-alanine supplementation is a popular ergogenic strategy. It should be noted that a substantial decrease in the HIS content (~30%) in muscles and plasma after beta-alanine supplementation has been reported [89]. However, in another, methodologically similar study, β-alanine supplementation for 28 days (6 g/day) did not reduce HIS in muscles [90]. Further studies are needed to determine whether beta-alanine supplementation requires a concomitant increase in HIS intake.

### 5.2. Effects on Neurodegenerative and Age-Related Disorders

It is now recognized that ROS/RNS and the neuronal histaminergic system contribute to the pathogenesis of neurodegenerative and age-related disorders, e.g., Parkinson’s and Alzheimer’s diseases, cancer, atherosclerosis, and cataract. Hence, HIS and HIS-containing substances may exert beneficial effects via their antioxidant, anti-inflammatory, and chelating properties and may modulate the histamine content in the brain.

In rats, HIS administration ameliorates aspirin-induced gastric mucosal damage [97], mitigates the development of brain infarction induced by the occlusion of the middle cerebral artery [98], and prevents isoproterenol- and doxorubicin-induced cardiotoxicity [99,100]. CAR administration rescues cognitive decline in a mouse model of Alzheimer’s disease [101]; suppresses tumorigenesis in human glioblastoma, pheochromocytoma, colorectal and ovarian carcinoma cells [102,103,104,105]; and delays the development of cataracts in diabetic rats [106].

Unfortunately, the articles reporting data from the clinical trials performed to date (Table 3) are rare and have various limitations, particularly because only a small number of subjects were evaluated, and sometimes, the results have been presented by only one research group.

### 5.3. Metabolic Syndrome

Metabolic syndrome refers to the cooccurrence of several risk factors, including insulin resistance, obesity, dyslipidaemia, and hypertension. It identifies a subgroup of patients who are at high risk of developing cardiovascular diseases and type 2 diabetes [112]. General characteristics of metabolic syndrome include oxidative stress and increased production of inflammatory cytokines, ROS/RNS, and AGE/ALE. Therefore, the anorectic effect of HIS supplementation and the anti-inflammatory and antioxidant properties of HIS and CAR may be beneficial.

HIS or CAR supplementation has been shown to be effective on insulin resistance, plasma lipid levels, and inflammatory markers and has delayed the development of atherosclerosis in several rodent models of diabetes and metabolic syndrome [113,114,115,116]. The results of studies investigating the effects of HIS or CAR intake on metabolic syndrome in human subjects are summarized in Table 4.

### 5.4. Rheumatoid Arthritis

A significant decrease in the blood HIS concentration has been observed in patients with rheumatoid arthritis [121,122]. The cause is obscure. A randomized double-blind trial did not show an advantage of oral HIS over the placebo [1].

### 5.5. Inflammatory Bowel Disease

It has been shown that inflammatory bowel diseases, such as Crohn’s disease and ulcerative colitis, might be influenced by HIS administration. Orally administered HIS ameliorates murine colitis and suppresses the production of various inflammatory factors by macrophages [123]. A zinc-CAR complex was shown to protect the gastric mucosa from experimental ulcerations and Helicobacter pylori-associated gastritis [5,124]. Furthermore, it has been shown that a decreased plasma HIS level predicts a risk of relapse in patients with ulcerative colitis [125].

### 5.6. Organ Preservation for Transplantation and Myocardial Protection in Cardiac Surgery

The unique proton-buffering capability prompted the use of HIS as a component of solutions for the preservation of organs intended for transplantation [12,13,14]. A high HIS concentration of 198 mM is in the histidine-tryptophan-ketoglutarate (HTK) solution routinely used for myocardial protection in cardiac surgery [12]. In our recent study [39], the administration of a HIS load in a dose corresponding to the HIS load in human cardiac surgery to rats markedly increased ammonia levels and impaired the energy status of the liver and skeletal muscle.

### 5.7. Modulation of the Sensitivity of Cancer Cells to Methotrexate

Methotrexate is a widely used anticancer agent that inhibits dihydrofolate reductase, an enzyme that generates tetrahydrofolate, an essential cofactor in nucleotide synthesis. A depletion of THF causes cell death by suppressing DNA and RNA synthesis. It has been suggested that the drain of cellular pool of THF by dietary HIS supplementation might improve methotrexate efficacy and might enable reduced dosing of this toxic agent [126].

### 5.8. Atopic Dermatitis

Atopic dermatitis (eczema) is a chronic inflammatory disorder characterized by itchy, red, and cracked skin. The aetiology has been linked to deficiencies in the HIS-rich epidermal barrier protein termed filaggrin [127]. It can be assumed that observations of eczematous rash in infants and adults fed a HIS-deficient diet [27] are due to impaired filaggrin formation.

Studies performed in rodents revealed the rapid incorporation of ^3^ H-HIS filaggrin after an injection [128], and in vitro studies using human keratinocytes showed that HIS increases filaggrin protein formation [3]. Data from one clinical study performed on 24 adults revealed that 4 g of HIS administered once daily over a period of 4 weeks improved symptoms of eczema [3].

### 5.9. Anaemia of Patients with Uraemia

A HIS deficiency may contribute to the anaemia observed in uremic patients since HIS is essential for haemoglobin synthesis; furthermore, a HIS-deficient diet is associated with anaemia development [27]. Anaemia associated with decreased HIS concentration in the blood has been repeatedly observed in patients with chronic kidney disease, particularly in patients undergoing dialysis [129,130]. Increased haematocrit values in HIS-supplemented patients with uraemia have been reported by Giordano et al. [2]. However, the design of the study by Giordano and colleagues has been criticized by Phillips et al. [131], and the results of 2 subsequent studies suggested that HIS supplementation did not improve anaemia in patients with uraemia [129,130].

The use of recombinant human erythropoietin therapy and iron supplementation almost completely eradicated severe anaemia in uremic patients, and therefore, the potential benefits of HIS therapy appear to be obsolete. However, unfortunately, some haemodialysis patients have anaemia that is refractory to erythropoietin therapy, and several pharmacological agents, such as androgens, vitamin C, and L-carnitine, have been studied to determine their effects on improving the response to erythropoietin [132]. Studies examining the effects of HIS supplementation on these patients are not available.

In addition to the supposed positive effect of HIS on erythropoiesis, a reason to advocate HIS supplementation in patients with chronic kidney disease is its potential to neutralize excessive production of ROS and tissue damage associated with iron overload [133]. Combined supplementation of iron with HIS has been shown to be more effective in therapy of uremic anemia when compared with iron alone [129].

## 6. Side Effects of Increased HIS Intake

There are no reports of signs of toxicity or mutagenic activity in HIS-treated subjects, and researchers have reached a consensus that increased intake of HIS and/or CAR is safe [134]. Although HIS is a precursor of histamine, allergic reactions or peptic ulcers caused by increased gastric acid secretion have not been reported. Practically important might be reduced folate status [45,46,126], anorexia [62,63,64,65,66,67,68,69], and increased loss of zinc in urine reported after HIS administration in overweight subjects and patients with progressive systemic sclerosis [135,136]. Several metabolic alterations noted below indicate that increased HIS consumption is inappropriate in subjects with liver injury.

First, the results of several studies performed using rats reveal that a HIS-supplemented diet may induce hypercholesterolemia and liver enlargement [137,138,139,140].

Second, 3 nitrogen atoms are present in the HIS molecule, which should appear as ammonia when HIS is catabolized. A high ammonia concentration in HIS-loaded subjects, which might occur after an infusion of HTK solution during cardiac surgery, may exert detrimental effects on the course of the underlying disease, particularly in subjects with impaired hepatic function. Increased ammonia concentrations have been observed in blood plasma, liver, and muscles of HIS-loaded rats [39].

Third, several studies have shown that HIS administration may lead to marked alterations in aminoacidaemia, which may impair signs of hepatic encephalopathy. Increases in glutamate, alanine, and glutamine concentrations and decreases in glycine and branched-chain amino acid (BCAA; valine, leucine, and isoleucine) concentrations in blood plasma have been reported frequently [39,59,140,141,142,143].

## 7. Summary and Conclusion

HIS possesses unique chemical and metabolic properties that are the basis for its use as a treatment for a wide range of conditions. HIS-rich solutions have clear benefits in the preservation of organs for transplantation and myocardial protection in cardiac surgery. Further studies are needed to elucidate the effects on muscle fatigue during strenuous exercise, neurological disorders, metabolic syndrome, atopic dermatitis, uraemic anaemia resistant to erythropoietin therapy, and inflammatory bowel diseases and as a supplement to increase the effectiveness of methotrexate in treatment of malignancies.

Signs of toxicity, mutagenic activity, and allergic reactions have not been reported. Of concern should be reports of hepatic enlargement, increases in ammonia and glutamine levels, and decreases in BCAA levels, indicating that HIS supplementation might be inappropriate in patients with liver disease.

In conclusion, HIS-containing supplements appear to be safe and efficient compounds with a promising therapeutic potential in remarkably large number of conditions. Randomized controlled intervention trials in humans utilizing HIS-containing compounds are warranted to validate their effectiveness for specific disorders.

## Figures and Tables

**Table 1:** Histamine receptors.

Receptor	Expression	Main Functions
H1	Ubiquitously (brain, respiratory epithelium, endothelial and smooth muscle cells, and lymphocytes)	Causes bronchoconstriction and vasodilation (urticaria) and induces wakefulness in the brain.
H2	Gastric parietal cells, smooth muscle, brain, and heart.	Stimulates parietal cells to produce hydrochloric acid and vasodilation.
H3	Exclusively in neurons	Presynaptic receptor that inhibits the release of histamine from histaminergic neurons. Activation promotes sleep.
H4	Immune cells, mast cells, intestinal epithelial cells, sensory neurons, and cancer cells	Induces chemotaxis and degranulation of mast cells.

**Table 2:** HIS-rich proteins and peptides.

HIS-Rich Protein Or Peptide	The Role	Reference
Haem-containing proteins (haemoproteins)	Structure of haemoglobin, myoglobin, cytochromes, haem peroxidases, nitric oxide synthase, catalases, etc.	[76]
HIS-rich glycoprotein	Plasma protein that interacts with many ligands, including zinc, phospholipids, fibrinogen, heparin, and immunoglobulins, plays roles in regulating several biological processes, such as coagulation and immunity.	[15]
Histatins	Salivary copper- and zinc-binding peptides with antibacterial, antifungal, and wound-healing properties. Investigated for the treatment of oral diseases.	[77]
HIS-rich calcium-binding protein	170 kDa protein primarily expressed in striated muscles and arteriolar smooth muscle cells with high capacity binding Ca^++^. Roles in the uptake, storage, and release of calcium ions by cardiac sarcoplasmic reticulum and regulation of cardiac rhythmicity.	[78]
Filaggrin (filament-aggregating protein)	Skin barrier protein that aggregates cytokeratin filaments of keratinocytes to form corneocytes. Degradation of filaggrin into amino acids, urocanic acid, and pyrrolidine carboxylic acid contributes to the formation of the “natural moisturizing factor” of the skin.	[40]

**Table 3:** Effects of HIS and HIS-containing dipeptides (HIS-CD) on the elderly and ageing-related disorders.

Study Design	Main Findings	Reference
Elderly volunteers (*n* = 39), anserine/CAR (3:1), 1 g/day, 3 months. A double-blind randomized controlled trial.	Positive effects on verbal episodic memory, decreased the secretion of proinflammatory cytokines, and improved brain perfusion.	[7]
Age-related cataract (*n* = 75), eye drops containing N-acetylcarnosine. Two drops, twice daily, for 9 months.	Rejuvenation of visual functions	[6]
Alzheimer’s disease, a mixture of antioxidants including CAR (100 mg/day) plus donepezil or a placebo plus donepezil for 6 months. A double-blind study.	Improvement of cognition functions.	[107]
Parkinson’s disease (*n* = 36), inclusion of CAR (1.5 g/day for 30 days) in the therapy.	Improvement in neurological symptoms and a decrease in blood plasma protein carbonyl and lipid hydroperoxide levels.	[8]
Gulf War illness (*n* = 25), CAR (500, 1000, and 1500 mg doses increasing at 4-week intervals) for 12 weeks. A double-blind randomized controlled trial.	Positive effect on cognitive functions.	[108]
Schizophrenia, administration of CAR as an adjunct treatment (2 g/day) for 3 months. A double-blind randomized controlled trial.	Improvement in the performance on cognitive tests.	[109]
Mental fatigue and sleep disruption (*n* = 20), HIS (1.65 g/day) for 2 weeks. A placebo controlled double-blind crossover trial.	Ameliorated feelings of fatigue and improved attentiveness and performance during working memory tasks.	[34]
Mental fatigue (*n* = 48), ingestion of dried bonito broth (2.45 g) for 4 weeks. A placebo controlled double-blind crossover trial.	Improved the mood state and increased performance on a simple calculation task.	[37]
Healthy females (*n* = 31), ingestion of dried bonito broth (4.5 g) for 2 weeks. A placebo controlled double-blind randomized crossover study.	Improved mood, increased peripheral blood flow, and decreased levels of urinary oxidative stress markers.	[36]
Elderly people (*n* = 56), anserine/CAR (2.5 g/day) for 13 weeks. Double blind study.	Decrease in the body mass index and improvement in cognitive functions and physical capacity.	[110]
Chronic heart failure (*n* = 50), CAR (500 mg/day orally) for 6 months. Prospective, randomized study.	Beneficial effects on exercise performance and quality of life.	[111]

**Table 4:** The effects of HIS and CAR on humans with obesity and metabolic syndrome.

Study Design	Main Findings	Reference
Subjects with prediabetes (*n* = 62) and supplement containing cinnamon, chromium, and CAR (200 mg/day), 4 months. Double-blind, placebo-controlled study.	Decrease in fasting plasma glucose levels and increase in the fat-free mass.	[117]
Obese women with metabolic syndrome, HIS (4 g/day), 12 weeks. Double-blind, placebo-controlled study.	Improved insulin sensitivity and decreased body mass index, waist circumference, body fat, and markers of systemic inflammation.	[87]
Examination of serum HIS concentrations in obese (*n* = 235) and non-obese (*n* = 217) women.	Lower HIS concentrations were observed in obese women than in nonobese; negative relationships with inflammation and oxidative stress were identified.	[118]
Examination of HIS and energy intake by female Japanese students (*n* = 1689) aged 18 years.	Daily HIS intake correlated inversely with energy intake.	[119]
Internet-based cross-sectional study in a Chinese population (*n* = 88).	Dietary HIS intake was inversely correlated with energy intake, the status of insulin resistance, inflammation, oxidative stress, and the prevalence of obesity.	[120]
